# The Synergistic Effects of SHR6390 Combined With Pyrotinib on HER2+/HR+ Breast Cancer

**DOI:** 10.3389/fcell.2021.785796

**Published:** 2021-12-16

**Authors:** Yukun Wang, Xiang Yuan, Jing Li, Zhiwei Liu, Xinyang Li, Ziming Wang, Limin Wei, Yuanpei Li, Xinshuai Wang

**Affiliations:** ^1^ Henan Key Laboratory of Cancer Epigenetics, Cancer Hospital, The First Affiliated Hospital, College of Clinical Medicine, Medical College of Henan University of Science and Technology, Luoyang, China; ^2^ UC Davis Comprehensive Cancer Center, Department of Internal Medicine, University of California, Davis, Davis, CA, United States

**Keywords:** CDK 4/6 inhibitor, HER-2 inhibitor, HER2+/HR+ breast cancer, synergy, FoxM1

## Abstract

HER2+/HR+ breast cancer is a special molecular type of breast cancer. Existing treatment methods are prone to resistance; “precision treatment” is necessary. Pyrotinib is a pan-her kinase inhibitor that can be used in HER2-positive tumors, while SHR6390 is a CDK4/6 inhibitor that can inhibit ER+ breast cancer cell cycle progression and cancer cell proliferation. In cancer cells, HER2 and CDK4/6 signaling pathways could be nonredundant; co-inhibition of both pathways by combination of SHR6390 and pyrotinib may have synergistic anticancer activity on HER2+/HR+ breast cancer. In this study, we determined the synergy of the two-drug combination and underlying molecular mechanisms. We showed that the combination of SHR6390 and pyrotinib synergistically inhibited the proliferation, migration, and invasion of HER2+/HR+ breast cancer cells *in vitro*. The combination of two drugs induced G1/S phase arrest and apoptosis in HER2+/HR+ breast cancer cell lines. The combination of two drugs prolonged the time to tumor recurrence in the xenograft model system. By second-generation RNA sequencing technology and enrichment analysis of the pyrotinib-resistant cell line, we found that FOXM1 was associated with induced resistance to HER2-targeted therapy. In HER2+/HR+ breast cancer cell lines, the combination of the two drugs could further reduce FOXM1 phosphorylation, thereby enhancing the antitumor effect to a certain extent. These findings suggest that SHR6390 combination with pyrotinib suppresses the proliferation, migration, and invasion of HER2+/HR+ breast cancers through regulation of FOXM1.

## Introduction

According to the Cancer Research Institute, breast cancer is the most frequently diagnosed malignant tumor, ranking the first among all cancers causing death in women. It is reported that the incidence of breast cancer continues to rise. In 2020, there were an estimated 2,261,419 new cases of breast cancer worldwide, accounting for 11.7% of all new cancer patients and 24.5% of new cancer patients in women (International Agency for Research on Cancer, 2020). Because breast cancer is clinically and histologically heterogeneous, there are four molecular subtypes from the St. Gallen Consensus 2013: Luminal A-like, Luminal B-like (HER2 positive; HER2 negative), Erb-B2 overexpression, and Basal-like. Each subtype requires different diagnostic and therapeutic approaches. Luminal B-like (HER2 positive), also HER2+/HR+ breast cancer, is recommended for combined treatment of cytotoxicity, anti-HER2, and endocrine therapy (Goldhirsch et al., 2013; Cho, 2016). However, HER2+/HR+ breast cancer was less endocrine sensitive and more aggressive and had worse prognosis compared with HR+/HER2-breast cancer (Lipton et al., 2002; De Laurentiis et al., 2005; Dowsett et al., 2008). It is necessary to design personalized treatment strategies by considering the heterogeneity of HER2+/HR+ breast cancer diseases (Collins and Varmus, 2015; Ashley, 2016).

**GRAPHICAL ABSTRACT:**
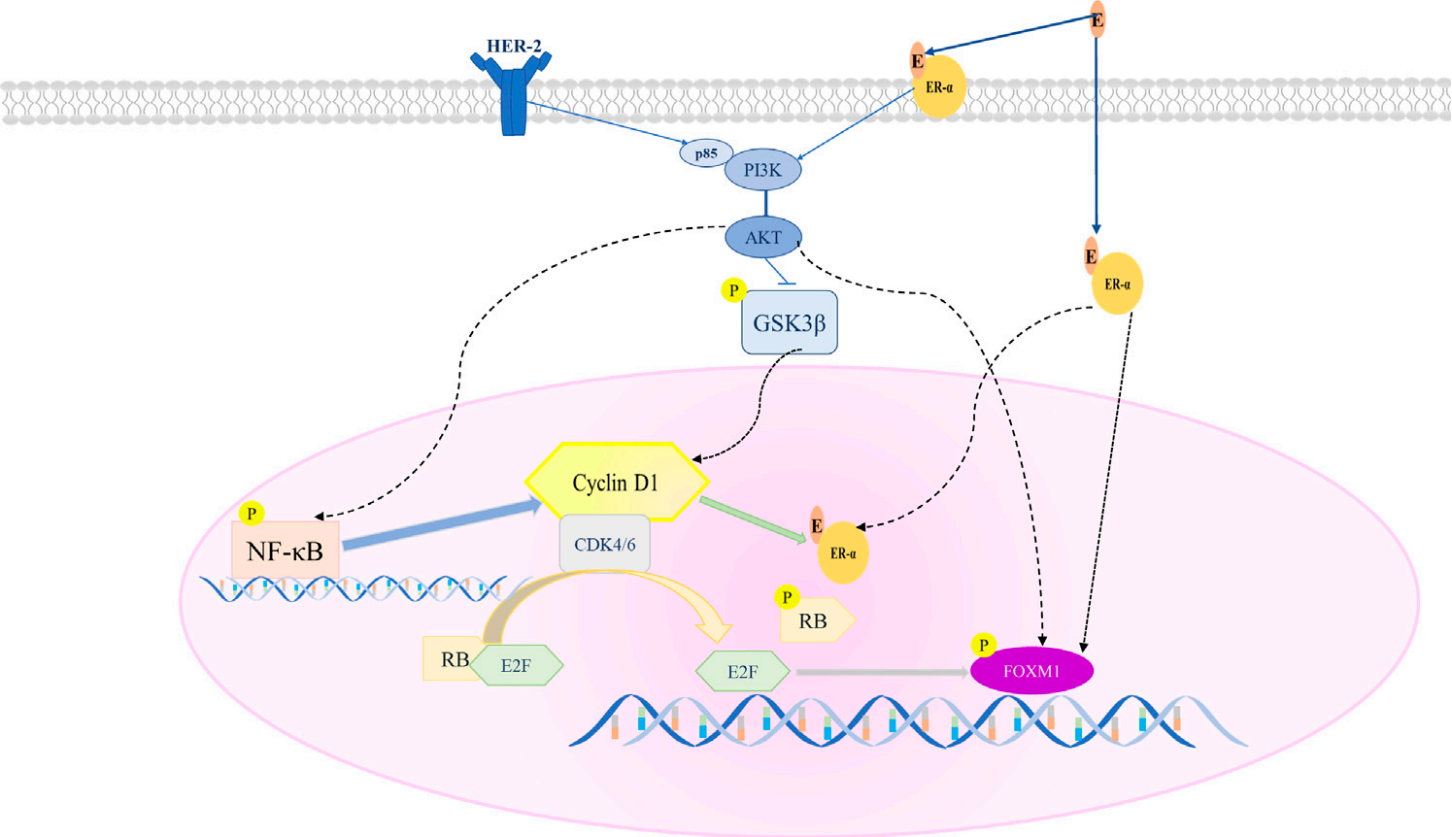
A proposed model of Pyrotinib combined with SHR6390 to inhibit HR+/HER2+ breast cancer proliferation. SHR6390 combined with Pyrotinib inhibits the downstream signal FOXM1, and has a significant effect on cell proliferation, migration and invasion of HR+/HER2+ breast cancer.

Treating HER2+/HR+ breast cancer is complicated. HER2+/HR+ tumors are usually effective for hormone therapy and/or HER2-targeted therapy at first, but they develop resistance over time. This is because in the entire treatment process, pathway interaction and mutual interference may lead to changes in the course of cancer. One of the reasons may be the known cross talk between the ER and HER2 signaling pathways. Intracellular signal transduction of estrogen includes nuclear genomic activity initiated by nuclear and non-nuclear genomic activity initiated by membrane. Non-nuclear genomic activity interacts directly or indirectly with the HER2/HER1-4 dimer to activate its downstream kinase pathways (such as Ras-MAPK and PI3K-Akt pathways) and phosphorylate ER to make cells proliferate. A study on HER2+ cell lines showed that after receiving lapatinib and trastuzumab treatment, ER or its downstream signals were significantly upregulated (Wang et al., 2011). A study showed that ER- were converted to ER+ in some HER2+ tumors following 2 weeks of neoadjuvant lapatinib treatment (Giuliano et al., 2015). Therefore, the HER2 pathway may lead to increased phosphorylation of ER and promote resistance to endocrine therapy (Schettini et al., 2016). In addition, studies have shown that membrane ER has cross talk with EGFR and IGFR-1 signaling pathways and is involved in the development of drug resistance to targeted HER2 therapy (Miller et al., 2011). Studies have shown that HER2 genome amplification of circulating tumor DNA was associated with ER positivity and/or PR positivity in primary resistance to T-DM1 HER2+ breast cancer (Sakai et al., 2018). Therefore, there is currently no treatment method or combination of treatments that is completely suitable for this subtype of patients. Analyzing the biological drivers of cancer can help to explore effective treatment strategies (Kay et al., 2021).

Studies have shown that Cyclin D is essential for tumor maintenance (Choi et al., 2012), and it is overexpressed in most cases of breast cancer (Deshpande et al., 2005; Musgrove et al., 2011). ER+ breast cancer is largely dependent on estrogen signals (Varma et al., 2007). ER signaling mainly upregulates cyclin D1 levels, especially culminating in the upregulation of CDK4/6 activity (Watts et al., 1994; Foster et al., 2001). ER inhibition can lead to cell cycle arrest in the G1 phase (Sutherland et al., 1983; Carroll et al., 2000). Besides, cyclin D1 can independently activate ER. In summary, cyclin D-CDK4/6-mediated signaling has a potential role in estrogen-independent ER+ breast cancer (Dean et al., 2010). Furthermore, it is shown that mice lacking functional cyclin D1 are resistant to cancer caused by the ErbB-2/HER2/neu oncogene (Landis et al., 2006). Moreover, studies on adult female mice bearing ErbB2-driven breast tumors have shown that inhibition of cyclin D-CDK kinase activity can trigger tumor cell senescence and prevent the progression of breast cancer (Choi et al., 2012). Therefore, the two pathways of ER and HER2 may converge and eventually exert its downstream effect on the cyclin D-CDK4/6 pathway. Furthermore, it is reported that the amplification of cyclin D1 and CDK4 is particularly high in HER2+/HR+ breast cancer (58 and 25%, respectively). Therefore, targeting CDK4/6 is a very reasonable strategy in HER2+/HR+ breast cancer (Finn et al., 2016).

Pyrotinib is a new generation of HER2 small-molecule tyrosine kinase inhibitor with anti-EGFR HER2 and HER4 activity (Waks and Winer, 2019). It can prevent the formation of homogeneity and heterodimer of HER2 and EGFR in tumor cells and inhibit its own phosphorylation and block the activation of downstream signaling pathways, thereby inhibiting tumor cell growth (Blair, 2018). SHR6390 is a CDK4/6 inhibitor. At present, preclinical or clinical studies have not found that a single CDK4/6 inhibitor can successfully inhibit HER2+/HR+ breast cancer. Therefore, we hypothesize that the two signaling pathways could be nonredundant and the co-inhibition of both pathways by combination of SHR6390 and pyrotinib may have synergistic anticancer activity on HER2+/HR+ breast cancer *in vivo* and *in vitro*. Here we determined the synergy of the two drugs with our *in vitro* and *in vivo* mouse models and explored the potential mechanisms with RNA-seq.

## Materials and Methods

### Cell Lines and Cell Cultures

Human HER2+/HR+ breast cancer cell lines: BT474 was purchased from the American Type Culture Collection (ATCC, Manassas, VA, United States). EFM-192A was purchased from Shanghai Cell Bank (Shanghai, China). EFM-192A cells were maintained in DMEM medium supplemented with 10% FBS. BT474 was cultured in RPMI 1640 supplemented with 10% FBS.

### Chemicals and Antibodies

Pyrotinib (SHR 1258) and SHR6390 were obtained from Jiangsu Hengrui Medicine (Jiangsu, China). Agents were dissolved in dimethylsulfoxide (DMSO) at a concentration of 20 mM and were then kept at −20°C until further use. Antibodies were purchased from Bioss (Beijing, China): GSK-3β, GSK-3β (Ser9), NF-κB, pNF-κB (Ser468), FOXM1, RB, pRb (Ser 780); pFOXM1 was purchased (Thr600) from Affinity Biosciences. Antibodies against GAPDH were purchased from Cell Signaling Technology (Beverly, MA, United States).

### Cell Viability (MTT Assay) and Drug Combination Study

We determined the 50% inhibitory concentration (IC50 value) of different cell lines through MTT assay. The cells are seeded into a 96-well plate at a density of 3,000–10,000 cells/well. The next day, we treated the cells with pyrotinib, SHR6390, the combination of the two drugs, or DMSO; the treatment continued for 72 h. The microplate reader (BioTek, Norcross, GA, United States) was operated to automatically detect the optical density value (OD value) of each well. We took the average of the OD values (absorbance) of each group of eight wells and calculated the cell inhibition rate of each group. IR (inhibition rate) = (1-OD value of the experimental group/OD value of the negative control group) × 100% (Van Meerloo et al., 2011). GraphPad software (GraphPad Prism 5) was used to calculate the IC50 value, after the cell inhibition rate was obtained. CompuSyn (ComboSyn Inc.) was used to calculate the Combination Index (CI) value (Chou, 2018). The CI value can show the effect of synergistic (<1), additive (=1), or antagonistic (>1) effect of the two-drug combination.

### Pyrotinib-Resistant HER2+/HR+ Breast Cancer Cell Line Induction

The pyrotinib-resistant HER2+/HR+ breast cancer cell line was inducted by gradually increasing cytotoxic drugs. The EFM-192A cell line was inducted from 10% IC50 under the action of pyrotinib. The cells were initially seeded in a Petri dish so that the confluence was about 20%. After 24 h, pyrotinib was added to the culture medium in the universal container (initial concentration is 10% IC50 value), and then the culture medium was added to the cells in the Petri dish under standard aseptic conditions. When the cells were confluent, the cells can be subcultured in the usual way. We performed two subcultures at this concentration, and we increased the drug concentration if the cell viability was normal (note: freeze a batch of cells for each subculture). We stopped to induce drug resistance until the drug concentration significantly slowed down cell growth.

### KEGG Pathway Enrichment Analysis and Protein–Protein Interaction Network Analysis

Genetic sequencing on drug-resistant cell lines and parental cell lines was performed. Statistical software R (version 3.6.1, https://www.r-project.org/) and packages of Bioconductor (http://www.bioconductor.org/) were applied for the significant analysis of differentially expressed genes (DEGs) between the two cell lines. The “limma” (linear models for microarray data) R package was used to screen the DEGs between parental EFM-192A cell line and pyrotinib-resistant EFM-192A cell line. The SAM (significance analysis of microarrays) with FDR (false discovery rate) < 0.01 and |log2 fold change (FC)| ≥ 2 were chosen as the cutoff criteria. KEGG pathway enrichment analysis of DEGs was performed to explore the critical pathways closely related to pyrotinib-resistant HER2+/HR+ breast cancer initiation. The “ggplot2” package and “pathview” package (version 1.24.0), which were based on Bioconductor, were used to make the KEGG pathway enrichment analysis visualization. The PPI network was used to identify the key genes which are involved in HER2+/HR+ breast cancer development at the interaction level. The screened DEGs were submitted to the STRING database (version 11). Then, Cytoscape software (version 3.7.1) was used for construction of the PPI network. MCODE, a plug in Cytoscape, was used for the visual analysis with “Combined score > 0.2 and MCODE > 11” as the threshold.

### Wound Closure Assay

The experimental groups were added with pyrotinib (20% IC50, 50% IC50), SHR6390 (20% IC50, 50% IC50), and the combination of the two drugs (50% IC50 pyrotinib and 50% IC50 SHR6390), and the no-drug group was the blank control group. The six groups of breast cancer cell lines were cultured in six-well plates. When the cell fusion degree was 80%, the cells were scratched with a 200-μl sterile pipette tip, washed twice with PBS, and added with serum-free medium for culture. Then, we used a Nikon TS100 microscope (Nikon, Tokyo, Japan) and randomly selected in each well three fields of view (×100) in the scratched area to take pictures at 0 h. The medium containing the drug and 10% fetal bovine serum was added, and after 24 h, each well was photographed in the abovementioned field of view. Measure the width of the scratch at different times, compare the difference in scratch healing between the groups, perform the test at least three times, and take the average value (Justus et al., 2014).

### Cell Invasion Assay

A suspension of 1 × 10^5^ HER2+/HR+ breast cancer cell lines was added to Transwell chambers (Corning Inc., Corning, NY, United States) with 50 µl of Matrigel (Beijing Solarbio Science & Technology Co., Ltd., Beijing, China). The experimental group was added with different concentrations of pyrotinib, SHR6390, and the combination of the two drugs, respectively. It was the vertical group without drugs. After 24 h of starvation, six groups of breast cancer cell lines were respectively added into the abovementioned 300-μl medium of different drug concentrations into the upper chamber of the Transwell chamber. The lower chamber is usually added with 500 μl medium containing 10% fetal bovine serum. After being cultured for 48 h, the cells in the upper chamber were gently scraped off with a sterilized cotton swab and stained with 0.1% crystal violet for 30 min. Then, the cells were taken out and rinsed in distilled water. The number of cells passing through the basement membrane of the ventricle was observed and counted under inverted microscopy (100× and 200× magnification) (Justus et al., 2014). The experiment was performed at least three times.

### Cell Cycle Analysis

Different concentrations of pyrotinib, SHR6390, and the combination of the two drugs were put to the experimental group, and the no-drug group was the blank control group. After 24 h of starvation, cells were trypsinized in the logarithmic growth phase, washed with cold PBS, fixed with absolute ethanol, and stored 1 h at −20°C. Then, the fixed cells were collected, washed, and resuspended with cold PBS. Then, the cell solution was adjusted to 5 × 10^5^ cells/ml, soaked in the RNase and propidium iodide (PI, Sigma-Aldrich, St. Louis, MO, United States) incubation solution for 15 min in the dark at 37°C, and finally analyzed by the flow method according to the manufacturer’s instructions. All experiments were performed three times independently (Nunez, 2001). Analysis was done by flow cytometry using FlowJo 10 software (Ashland, OR, United States).

### Apoptosis Analysis

The FITC Annexin V/PI assay was used to detect apoptosis. The experimental group was added with different concentrations of pyrotinib, SHR6390, and the combination of the two drugs, respectively. After 48 h of treatment, the cells were washed with PBS, adjusted to 5 × 10^5^ cells/ml, resuspended in binding buffer, and stained with FITC Annexin V (5 µl) (Annexin V-FITC Apoptosis Detection Kit, NJ, United States) and propidium iodide (5 µl). Then, the stained cells were incubated in the dark at 37°C for 30 min. Finally, the flow cytometer (BD FACSCalibur, BD Biosciences, San Jose, CA, United States) was used to evaluate cell counts to determine apoptosis, according to the manufacturer’s instructions (Riccardi and Nicoletti, 2006; Crowley et al., 2016). All analyses were in triplicate, independently.

### Western Blot Analysis

Cells were treated with different concentrations of drugs. Cells were lysed with cell lysis buffer, and PMSF, protease, and phosphatase inhibitors were added into the buffer. The proteins from the cells were separated by 10% SDS-PAGE and transferred to polyvinylidene fluoride (PVDF) membrane by electrophoresis. Proteins were visualized using a Western blot imaging system (Bio-Rad, Hercules, CA, United States) and then quantified using ImageJ software (National Institutes of Health, Bethesda, MD, United States).

### *In Vivo* Study (Xenograft Studies)

All experiments were conducted in accordance with the guidelines of the Regulations on the Management of Experimental Animals and were approved by the Experimental Animal Ethics Committee of Henan University of Science and Technology. Four-to five-week-old female BALB/c nu-mice were raised in the SPF laboratory breeding facility. The suspension of EFM-192A cells (1 × 10^7^ cells/100 μl) was injected into the skin of the left back of each mouse, 2 μl per mouse. The nude mice were fed routinely and observed every day. When the tumor reached 100–200 mm^3^, they were taken as the 0th day of medication. Mice were randomly assigned to four groups (seven in each group) and were treated by oral gavage with pyrotinib, SHR6390, and pyrotinib combined with SHR6390 vehicle (PBS) once a day. It was continuous for 25 days. During this period, tumors and weights of each mouse were measured twice a week. The tumor volume was calculated as V = 1/2 (length×width^2^). After the experiment was completed, the mice were executed humanitarianly. We collected tumor samples, recorded the tumor size, and calculated the multiplication rate. Samples of tumors were subjected to immunohistochemistry.

### Immunohistochemistry

Tumors were fixed in 4% formalin at room temperature, and then they were embedded in paraffin. The embedded paraffin was sectioned. Sections were stained with p-GSK3β, pNFκB, pFOXM1, and pRB antibodies.

### Statistical Analysis

GraphPad Prism Version 7 software (GraphPad software, San Diego, CA, United States) and IBM SPSS version 22 (SPSS, Armonk, NY, United States) were used for statistical analysis. The half-maximal inhibitory concentration (IC50) values were calculated by nonlinear regression analysis of the dose–response curve. We used ComboSyn Inc. software to calculate the CI values. The results were expressed as the means ± SD (standard deviation) of three independent tests. The analysis of variance model and Student’s *t* test were used to compare the differences between test groups. The statistical significance of the difference between the test sample and the control sample was evaluated when the significance threshold was *p < 0.05, **p < 0.01, ***p < 0.001.

## Results

### Combination of SHR6390 and Pyrotinib Synergistically Inhibits the Proliferation *In Vitro*

**FIGURE 1:**
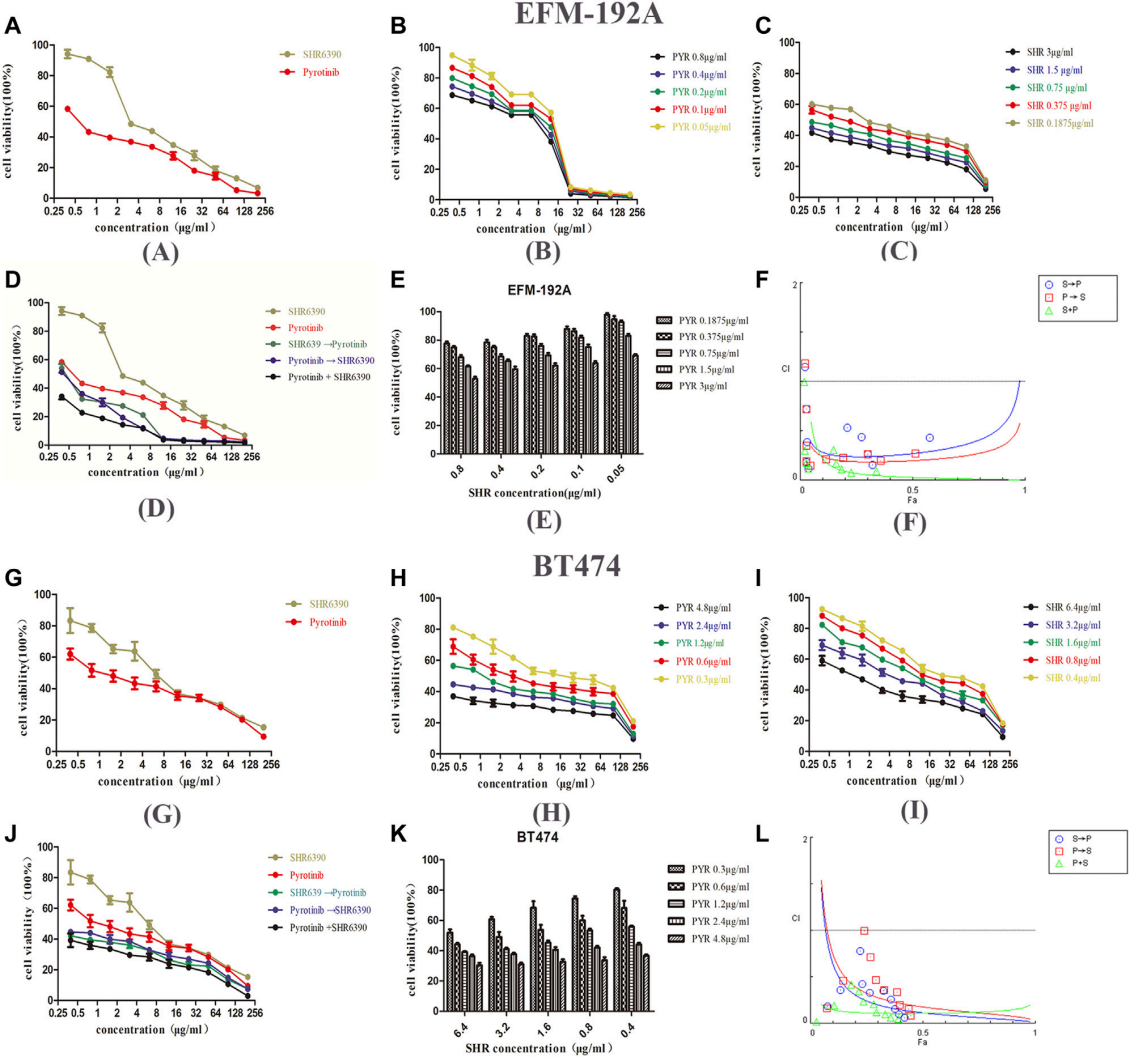
Effects of PYR and SHR on the viability of HER2+/HR+ breast cancer cell lines. **(1A)** Proliferation activity of the EFM-192A cell line was determined by the MTT assay after incubation for 48 h with different concentrations of PYR or SHR. **(1B**–**1D)** The EFM-192A cell line was treated with PYR or SHR alone or in combination or in sequences (PYR first for 6 h followed by SHR or SHR first for 6 h followed by PYR). **(1E)** Proliferation activity of the EFM-192A cell line was determined after incubation for 48 h with different concentrations of SHR combination with 0.1875, 0.375, 0.75, 1.5, and 3 μg/ml of PYR. **(1F)** The combination index (CI) vs. fraction-affected (Fa) affected plot was calculated by CompuSyn and depicted the combination effects. Synergistic growth inhibitory effects of pyrotinib (PYR) combined with SHR6390 (SHR) on the EFM-192A cell line. **(1G)** Proliferation activity of the BT474 cell line was determined by the MTT assay after incubation for 48 h with different concentrations of PYR or SHR. **(1H**–**1J)** The BT474 cell line was treated with PYR or SHR alone or in combination or in sequences (PYR first for 6 h followed by SHR or SHR first for 6 h followed by PYR). **(K)** The proliferation activity of the BT474 cell line was determined after incubation for 48 h with different concentrations of SHR combination with 0.3, 0.6, 1.2, 2.4, and 4.8 μg/ml of PYR. **(1L)** The combination index (CI) vs. fraction-affected (Fa) affected plot was calculated by CompuSyn and depicted the combination effects. Synergistic growth inhibitory effects of pyrotinib (PYR) combined with SHR6390 (SHR) on BT474 cell line.

Firstly, the inhibitory ability of SHR6390 and pyrotinib on EFM-192A and BT474 HER2+/HR+ breast cancer cell lines was evaluated. The results of the study showed that both SHR6390 and pyrotinib inhibited the tumor proliferation of HER2+/HR+ cell lines (pyrotinib IC50 0.8 ± 0.1 μg/ml in EFM-192A, 4.6 ± 0.2 μg/ml in BT474; SHR6390 IC50 3 ± 0.2 μg/ml in EFM-192A, 6.4 ± 0.2 μg/ml in BT474). Next, we determined whether the combination of SHR6390 and pyrotinib had a synergistic effect on HER2+/HR+ breast cancer cells. We evaluated the effect of different concentrations of pyrotinib and SHR6390 in inhibiting the proliferation of EFM-192A and BT474 cells (Figures 1A–E,G–K). The extension of the median effect equation (MEE) proves the combination index equation (CIE) (CI < 1) that defines the synergy, additive effect (CI = 1), and antagonism (CI > 1), and the data can be automatically simulated by the CompuSyn software, after it is entered into the software (Chou and Talalay, 1984). In order to determine whether the antitumor effects were synergistic, CompuSyn was used to calculate the CI value. The results showed that pyrotinib combined with SHR6390 significantly inhibited the growth of HER2+/HR+ breast cancer cell lines. In both cell lines, the CI value was less than 1, indicating that the two drugs had a synergistic effect (Figures 1F,L).

### FOXM1 is Associated With Inducted Resistance to HER2-Targeted Therapy

In order to explore the molecular mechanism of the combination SHR6390 and pyrotinib in synergistically inhibiting breast cancer cells, we induced EFM-192A cells with a low dose of pyrotinib. After 8 months of continuous induction, we obtained pyrotinib-resistant EFM-192A cell lines with 10 times the IC50 value. Then, next-generation RNA sequencing for the parental EFM-192A cells and the pyrotinib-resistant EFM-192A cells was performed.

**FIGURE 2:**
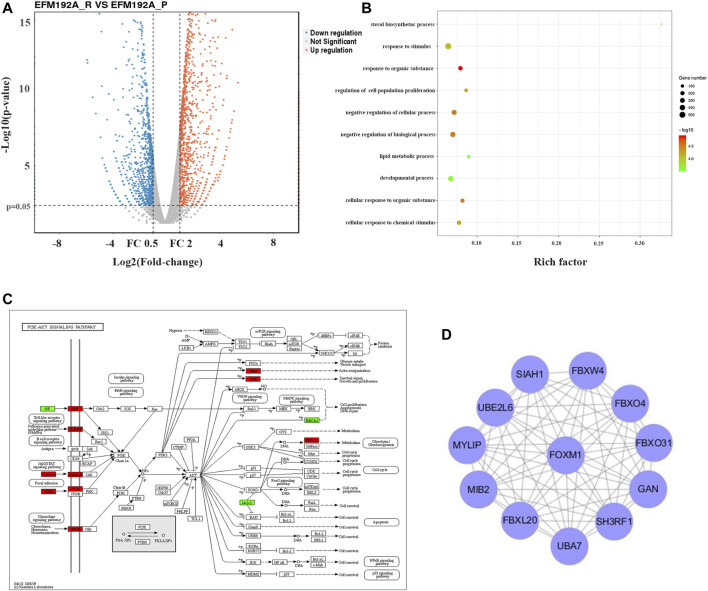
FOXM1 enriched was associated with induced resistance to anti-HER2 therapies. **(2A)** Volcano map with differential expression. The red lines represent upregulated genes, and the blue lines represent downregulated genes. **(2B)** GO enrichment analysis result of DEGs with |logFC| ≥ 2. **(2C)** Visualization of KEGG pathway enrichment of DEGs in parent and pyrotinib-resistant EFM-192A cell lines. **(2D)** Screen the hub genes from DEGs and PPI. DEG, differentially expressed gene.

After removing batch differences and data normalization, a total of 1,579 DEGs were obtained, including 819 upregulated genes and 760 downregulated genes based on cutoff criteria (*p* < 0.01 and |logFC| ≥ 2). All abnormally expressed genes with the log2 FC score and −log10 *p* value were used to generate a Volcano diagram in R, which is an intuitive tool to show the overall gene expression level of the DEGs (Figure 2A).

GO term enrichment analysis results varied from GO classification and expression change of DEGs. These significantly enriched pathways and terms could help us to further understand the role of DEGs in pyrotinib-resistant breast cancer occurrence and progress (Figure 2B). In addition, KEGG pathway enrichment analysis was used to examine the function of the DEGs. The result of KEGG analysis showed that the upregulated genes were significantly enriched in the PI3K-Akt signaling pathway (Figure 2C).

Using the STRING online database, 819 upregulated genes were filtered into the DEG PPI network complex containing nodes and edges with a combined score of >0.2. The Cytoscape software was employed to analyze the interactive relationship between the candidate proteins, and then a cluster containing 12 nodes was selected with a cutoff k-score = 11 by the MCODE scoring system. The results showed that the hub gene was the intersections between the initial key genes. FOXM1 enriched was associated with inducted resistance to anti-HER2 therapies (Figure 2D).

### Combined SHR6390 and Pyrotinib Synergistically Inhibit the Migration and Invasion of HER2+/HR+ Breast Cancer Cells *In Vitro*

**FIGURE 3:**
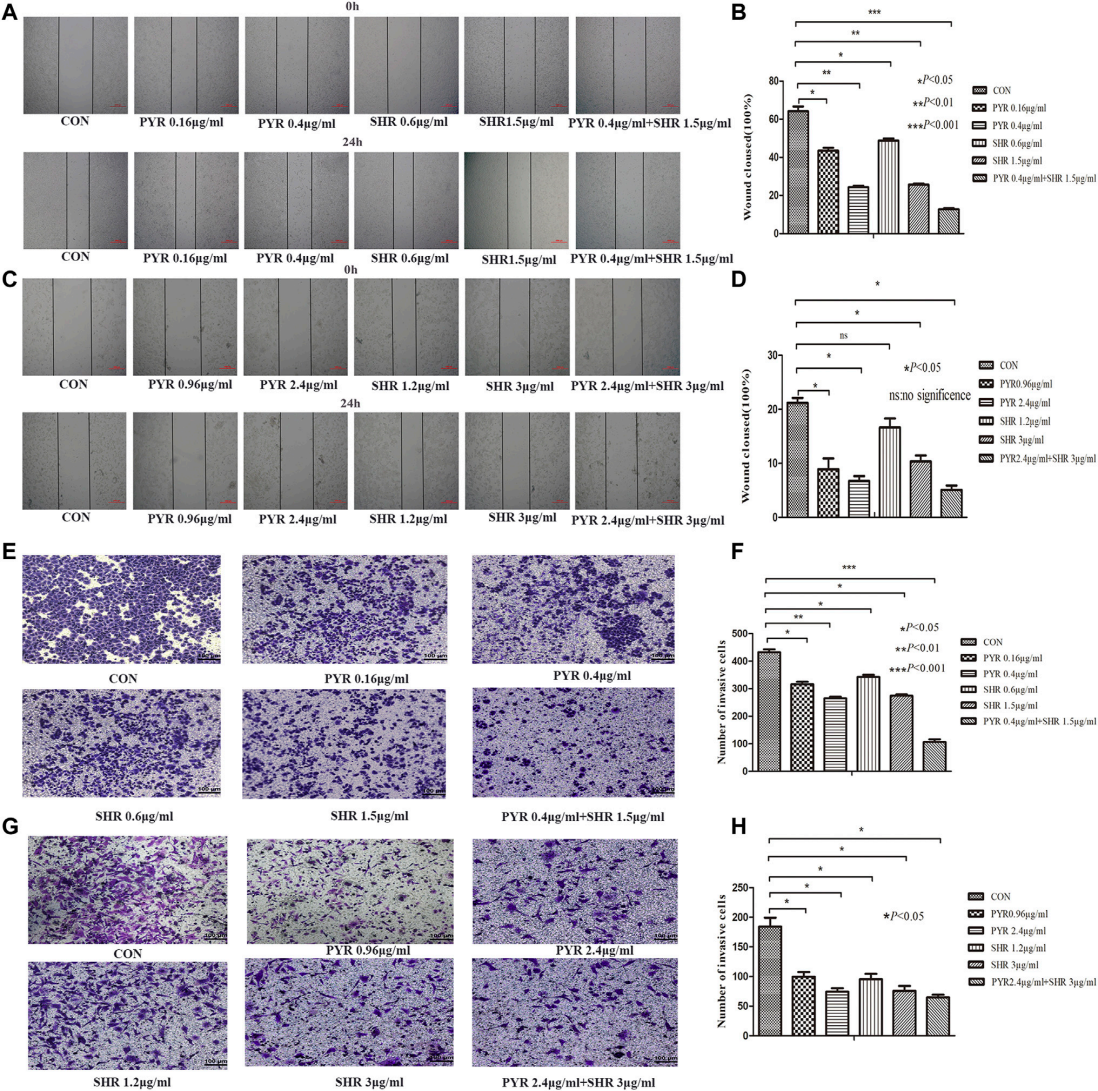
Effects of PYR and SHR on HER2+/HR+ breast cancer cell migration and invasion. **(3A)** Wound healing assay assessed the effect of PYR (0.16, 0.4 μg/ml), SHR (0.6, 1.5 μg/ml), or PYR (0.4 μg/ml) and SHR (1.5 μg/ml) on EFM-192A cells’ migration ability, and **(3B)** histogram represents the statistical analysis. **(3C)** Wound healing assay assessed the effect of PYR (0.96, 2.4 μg/ml), SHR (1.2, 3 μg/ml) or PYR (2.4 μg/ml), and SHR (3 μg/ml) on BT474 cells’ migration ability, and **(3D)** histogram represents the statistical analysis. Cell invasion was analyzed with a Matrigel-coated Boyden chamber. HER2+/HR+ breast cancer cell lines were treated with PBS(CON) and different concentrations of PYR and SHR for 24 h. **(3E)** Transwell invasion assays assessed the effect of PYR (0.16, 0.4 μg/ml), SHR (0.6, 1.5 μg/ml) or PYR (0.4 μg/ml), and SHR (1.5 μg/ml) on EFM-192A cell invasion ability, and **(3F)** histogram represents the statistical analysis. **(3G)** Transwell invasion assays assessed the effect of PYR (0.96, 2.4 μg/ml), SHR (1.2, 3 μg/ml) or PYR (2.4 μg/ml), and SHR (3 μg/ml) on BT474 cells’ invasion ability, and **(3H)** histogram represents the statistical analysis. Original magnification was ×100. Data represent the mean ± S.D. of three independent experiments. *p < 0.05, **p < 0.01, ***p < 0.001, ns: no significance, compared with the control.

The migration of HR+/HER2+ breast cancer cells decreased at 24 h in the pyrotinib or SHR6390 groups compared with the control group, and the migration rate of pyrotinib combined with SHR6390 was significantly reduced after 24 h. These results were consistent in both EFM-192A and BT474 cell lines. BT474 was a cell line with slow migration, and the control group could not close the wound after 24 h. A significant reduction in migration was shown after treatment with different concentrations of pyrotinib or SHR6390, while almost no migration was observed in the combination group. EFM-192A, a highly migratory cell line, almost completely closed the wound in the control group after 24 h. Different concentrations of pyrotinib or SHR6390 induced reduced wound healing at 24 h compared with untreated cells but were significant in the combination group (Figures 3A–D).

Quantitative analysis showed that the 10% FBS chemical attraction of cells in the vertical group resulted in the highest cell invasion rate. To evaluate the effect of pyrotinib or SHR6390 treatment, we compared the HER2+/HR+ breast cancer cell lines treated with different concentrations of SHR6390 and pyrotinib with the vertical group. We observed that the invasion rate decreased when cells were treated with pyrotinib or SHR6390; the higher the concentration, the less the cell migration. In the combination group of SHR6390 and pyrotinib, cell invasion decreased significantly. Similar results could be gotten in either the EFM-192A cell line or the BT474 cell line. All these results indicated that FBS chemotactic agents increased cell invasion ability, but this effect could be reduced by pyrotinib or SHR6390 treatment. In the combination of the two drugs, the invasion ability was significantly reduced. Therefore, pyrotinib combined with SHR6390 can weaken the invasion ability of HER2+/HR+ breast cancer cells (Figures 3E–H).

### The Combination of SHR6390 and Pyrotinib Induces G1/S Arrest and Apoptosis in HER2+/HR+ Breast Cancer Cell Lines

**FIGURE 4:**
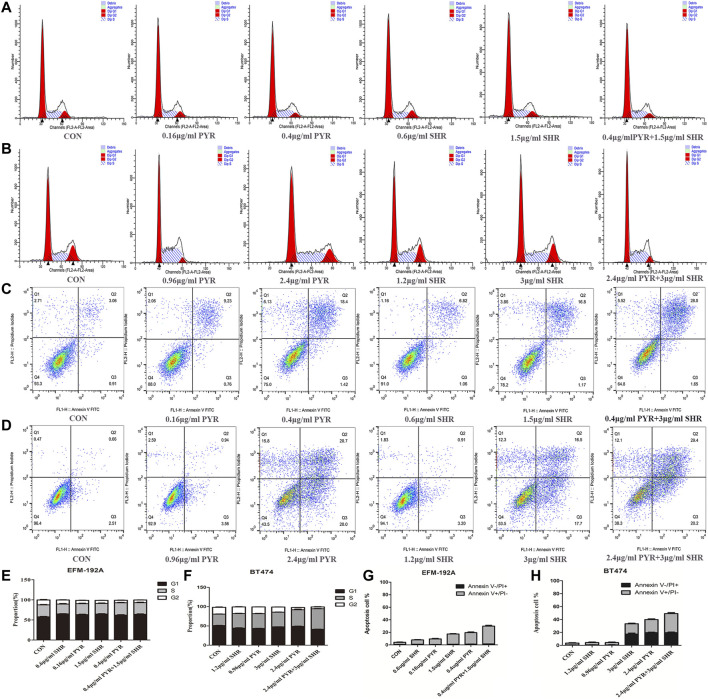
Effects of PYR and SHR on the cell apoptosis and cell cycle. **(4A)** Cell cycle analysis through PI staining and following flow cytometry for EFM-192A cells after incubated with PBS (CON), PYR (0.16, 0.4 μg/ml), SHR (0.6, 1.5 μg/ml) or PYR (0.4 μg/ml), and SHR (1.5 μg/ml) for 24 h. ModFit was used to perform cell cycle analysis. **(4B)** Cell cycle analysis through PI staining and following flow cytometry for BT474 cells after being incubated with PBS (CON), PYR (0.96, 2.4 μg/ml), SHR (1.2, 3 μg/ml) or PYR (2.4 μg/ml), and SHR (3 μg/ml) for 24 h. **(4E,4F)** The histograms were the representative results. Compared with control, PYR and SHR caused significant G1/S phase arrest. **(4C)** Cell apoptosis was detected through Annexin V-FITC/PI double staining and following flow cytometry for EFM-192A cells after being incubated with PBS (CON), PYR (0.16, 0.4 μg/ml), SHR (0.6, 1.5 μg/ml) or PYR (0.4 μg/ml), and SHR (1.5 μg/ml) for 24 h. **(4D)** Cell apoptosis for BT474 cells after being incubated with PBS (CON), PYR (0.96, 2.4 μg/ml), SHR (1.2, 3 μg/ml) or PYR (2.4 μg/ml), and SHR (3 μg/ml). **(4G,4H)** The histograms were the representative results. Compared to the control, PRY and SHR promote apoptosis and induces cellular stress in HER2+/HR+ breast cancer cell lines. Data represent the mean ± S.D. of three independent experiments.

The synergistic effect of SHR6390 and pyrotinib combined inhibition on cell viability could be mediated by changes in proliferation, apoptosis, or both. To explore this, the effect of SHR6390 and pyrotinib or combination therapy on cell-cycle progression was evaluated. It was found that in HER2+/HR+ breast cancer, both SHR6390 and pyrotinib could induce G1/S arrest. The combination of the two drugs could produce the greatest reduction in the percentage of G2 phase cells. The same conclusion was observed in both EFM-192A and BT474 cell lines (Figures 4A,B,E,F).

We also sought evidence of increased tumor cell apoptosis after combination therapy. For the two cell lines, we treated the cells with different concentrations of drugs (pyrotinib: 20% IC50, 50% IC50; SHR6390: 20% IC50, 50% IC50). It was found that both SHR6390 and pyrotinib could increase cell apoptosis. The greater the drug concentration we treated, the higher the apoptosis rate. Then, the combination of SHR6390 and pyrotinib was used to treat the cells, and the apoptosis rate of the two cell lines was further increased (Figures 4C,D,G,H).

Therefore, the enhanced inhibition of cell viability observed when combining SHR6390 and pyrotinib (compared to either drug alone) appears to be mediated by both increased suppression of cell proliferation and increased apoptosis.

### The Combination of SHR6390 and Pyrotinib Potentiates the Suppression of FOXM1 Phosphorylation

Western blot was used to assess the protein content of cell lines treated with SHR6390, pyrotinib, or the combination of the two drugs. We found that both SHR6390 and pyrotinib could reduce the phosphorylation of FOXM1, and the two drugs combined with FOXM1 significantly reduce the phosphorylation. It showed that the cyclin D‐CDK4/6 complex phosphorylates retinoblastoma proteins (RB) and dissociates them from the E2F transcription factors, which were ultimately responsible for cell-cycle progression (Pandey et al., 2019). Besides, both SHR6390 and pyrotinib could inhibit the phosphorylation of RB. The phosphorylation of RB dropped sharply after the combined treatment.

**FIGURE 5:**
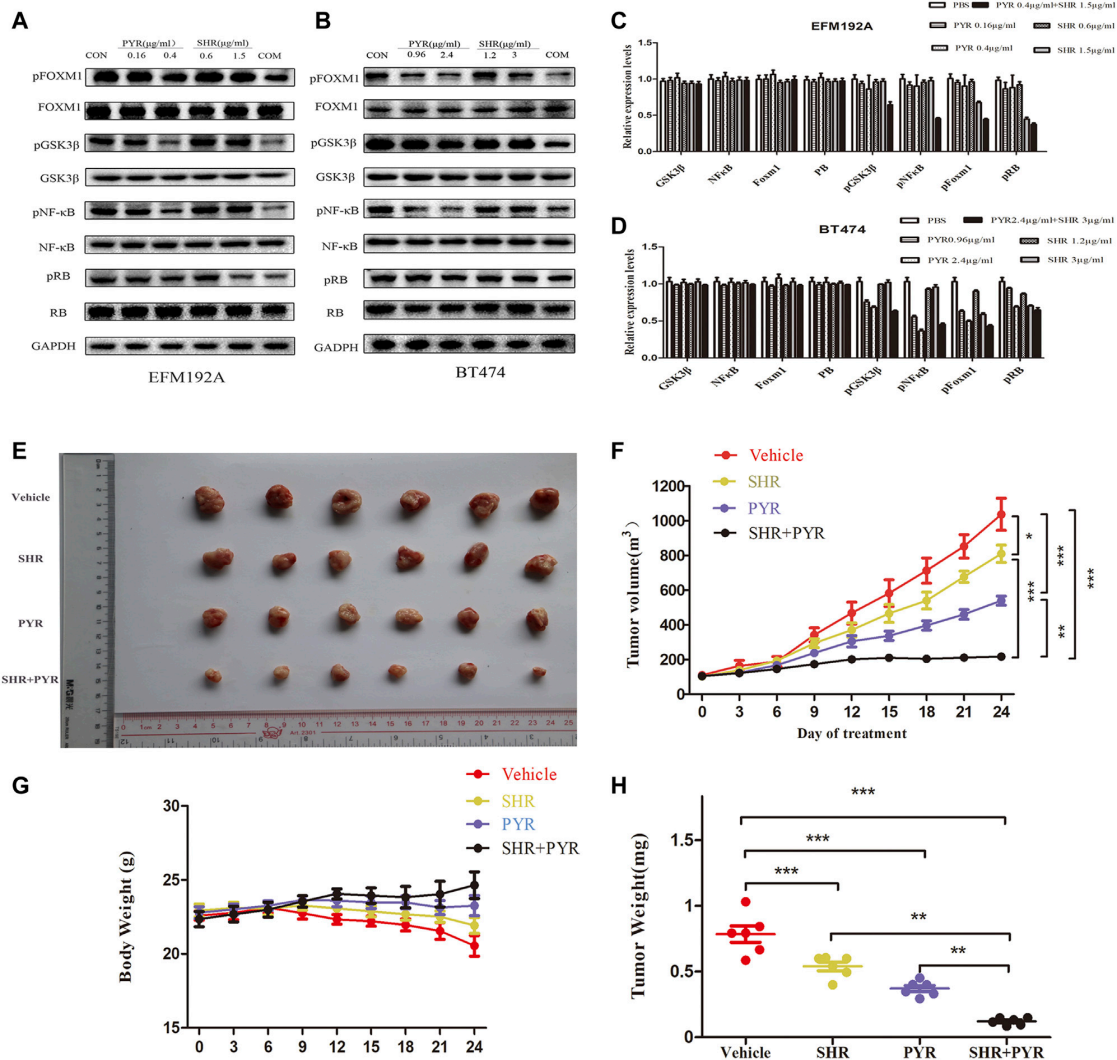
Molecular mechanism studies in HER2+/HR+ breast cancer cell lines after treatment and *in vivo* anticancer effect of PYR and SHR in cancer xenograft models. EFM-192A cells were treated with PBS, PBS (CON), PYR (0.16, 0.4 μg/ml), SHR (0.6, 1.5 μg/ml) or PYR (0.4 μg/ml), and SHR (1.5 μg/ml) for 24 h, respectively. BT474 cells were treated with PBS, PYR (0.96, 2.4 μg/ml), SHR (1.2, 3 μg/ml) or PYR (2.4 μg/ml), and SHR (3 μg/ml) for 24 h, respectively. Nuclear and cytosolic protein extracts were subjected to Western blot analysis. **(5A,5B)** The results of Western blot for FOXM1, pFOXM1, NFκB, pNFκB, GSK3β, pGSK3β, RB, and pRB in the nuclear fractions and cytosolic extracts, respectively. GAPDH served as the loading control. **(5C,5D)** Quantitative analysis of the Western blotting results. Data represent the mean ±S.D. of three independent experiments. Randomly grouped nude mice were treated with PBS (Vehicle), PYR (10 mg/kg/day), SHR (75 mg/kg/day), or a combination treatment (PYR+SHR) for 25 days. **(5E)** Photos of the excised tumors. **(5F,5G)** Tumor growth ratio curve and body weight changes every 3 days after the onset of treatment. **(5H)** Weight obtained on day 25 after treatment, *p < 0.05, **p < 0.01, ***p < 0.001 compared with the control.

In the PI3K-AKT pathway (Figure 2C), we found that GSK3 was related to CCND1 and the cell cycle. Studies showed that treatment with CDK4/6 inhibitors (such as palbociclib) can prevent RB phosphorylation and preserve the activity of NF-κB transcription (Kim et al., 2019). After treatment with SHR6390 and pyrotinib, through the detection of p-GSKβ protein expression, we found that pyrotinib significantly reduced the phosphorylation of GSKβ, but SHR6390 had no significant effect on it. The same conclusion can be obtained when detecting the phosphorylation of NFκB. In summary, we speculated that SHR6390-combined pyrotinib inhibited the proliferation of HER2+/HR+ breast cancer through FOXM1 (Figures 5A–D).

### SHR6390 and Pyrotinib Suppress the Growth of HER2+/HR+ Breast Cancer Xenografts

For evaluating the effect of pyrotinib and SHR6390 in HER2+/HR+ breast cancer *in vivo*, we tested whether these two drugs alone or in combination could inhibit tumor growth in a xenograft mouse model. The mice were transplanted with BT474, and they were randomly divided into four groups. These groups include the vehicle group, pyrotinib (10 mg/kg/day) (Jianing et al., 2021), SHR6390 (75 mg/kg/day) (Long et al., 2019), or the simultaneous administration of pyrotinib and SHR6390. We found that pyrotinib was more effective than SHR6390 in inhibiting the growth of BT474 tumors. However, compared with the two drugs alone, the combined use of pyrotinib and SHR6390 has a much stronger inhibitory effect on the growth of BT474 xenografts (Figures 5E,F). The weight of the combined treatment group was better than that of the single-drug group (Figures 5G,H). These data indicated that the combination therapy including SHR6390 and pyrotinib could enhance anticancer activity.

Immunohistochemical results showed that the expression levels of pFOXM1, pGSK-3β, pNF-κB, and pRB were low in both the control group and the experimental group. This result is inconsistent with that at the cellular level (Supplementary Figure S1). In our analysis, it might be due to the inactivation of phosphorylated protein during tumor tissue embedding and paraffin section processing, so no positive results were obtained. We need to further investigate the effect of FOXM1 on HER2+/HR+ breast cancer at the tissue level.

## Discussion

The treatment of HER2+/HR+ breast cancer has been controversial because some tumors in this luminal HER2+ subtype behave like luminal A cancer, while others behave like non-luminal HER2+ breast cancer. It is necessary to explore precise treatment options for subgroups of breast cancer with both HR+ and HER2+.

In this study, we found that the combination of CDK4/6 inhibitor SHR6390 and pan-her inhibitor pyrotinib in HER2+/HR+ cell lines (EFM-192A and BT474) shows synergistic inhibition of tumor proliferation and enhances antitumor effects *in vitro*. Our experimental data show that the combination of the two drugs inhibits the migration and invasion of HER2+/HR+ breast cancer cells *in vitro*. They block the cell cycle in the G1/S phase and increased tumor cell apoptosis, thereby reducing cells. The combination of SHR6390 and pyrotinib is effective *in vivo*.

Our results indicate that the anticancer effect of the combined therapy is higher than any other single drug and is consistent with that *in vitro*. In addition, nude mice received the combination therapy, but their body weight was not worse than those that received SHR6390 or pyrotinib alone. Therefore, our findings reveal that the combination of SHR6390 and pyrotinib exhibits synergistic antitumor activity without extended toxicity. It provides the possibility for clinical translation.

In addition, through our *in vitro* pyrotinib resistance induction experiment and subsequent RNA-seq analysis, we identified that FOXM1 is the intersection between the initial key genes. Our results show that both SHR6390 and pyrotinib can reduce activated FOXM1 phosphorylation, and the combination of two drugs could significantly reduce the phosphorylation further. We therefore propose that SHR6390 combined with pyrotinib inhibits the proliferation of HER2+/HR+ breast cancer through regulation of the FOXM1. Previous studies showed that FoxM1 is expressed in proliferating embryonic cells, but it is almost undetectable in most normal tissues (Wonsey and Follettie, 2005). Besides, it is reported that knocking out FOXM1 in breast cancer cells can restore sensitivity to endocrine therapy (Bergamaschi et al., 2014) and FOXM1 plays a role in the development of HER2-targeted therapy resistance (Lam et al., 2009). These and our study identify FOXM1 to be an attractive tumor-specific gene critical for breast cancer development and could be served as a potential therapeutic target for HER2+/HR+ breast cancer.

Through KEGG pathway analysis, we found that upregulated genes in pyrotinib-resistant cells are significantly enriched in the PI3K-Akt signaling pathway, which may lead to resistance to HER2-targeted therapies. It is consistent with previous research (Espin et al., 2015; Wilks, 2015). GSK3β is phosphorylated and inactivated downstream of the PI3K/Akt pathway. Akt phosphorylation of GSK3β can prevent the phosphorylation of cyclinD1, leading to the accumulation and nuclear localization of cyclinD1, the activation of CDK4/6, and the development of the cell cycle (Quintayo et al., 2012). In addition, studies have found that GSK-3β protects ERa from proteasome degradation and plays a key role in the stability and renewal of ERa protein (Grisouard et al., 2007).

NF-κB acts as a downstream effector of the growth factor pathway and participates in ligand-dependent and ligand-independent ER activation (Zhou et al., 2005). Studies have shown that NF-κB directly interacts with the DNA-binding function of ER through a variety of mechanisms (Franco et al., 2015). NF-κB can also affect ER by binding to the ER co-activator or co-inhibitor, which will change the ER transcriptional activity (Park et al., 2005). In addition, NF-κB directly binds to the cyclin D1 promoter and controls the transcription of cyclin D1 (Guttridge et al., 1999). Therefore, GSK-3β and NF-κB may be cross targets for HER2+/HR+ breast cancer. However, our findings suggest that pyrotinib significantly reduces the phosphorylation GSKβ, while SHR6390 has no significant effect on phosphorylation of GSKβ. The same conclusion can be obtained by detecting NFκB. These results show that pyrotinib inhibits the proliferation, migration, and invasion of breast cancer by regulating GSK-3β and NF-κB, regardless of the combination.

In summary, we show that SHR6390 and pyrotinib synergistically inhibit the proliferation of HER2+/HR+ breast cancer *in vitro* and *in vivo*. We reveal that the molecular mechanism for the combined effects is through regulation of FOXM1. Our study provides a theoretical basis for the comprehensive treatment of clinical breast cancer.

## Data Availability

The original contributions presented in the study are publicly available. These data can be found here: SRR16286554.

## Supplementary Material

Supplementary Figure 1 Immunohistochemical results showed that the expression levels of pFOXM1, pGSK-3β, pNF-κB and pRB about tumor tissue.
