# Supplementary figures and images for: The Synergistic Effects of SHR6390 Combined With Pyrotinib on HER2+/HR+ Breast Cancer
Source: Front Cell Dev Biol. 2021 Dec 16;9:785796. doi: 10.3389/fcell.2021.785796 (PMC8716872; doi:10.3389/fcell.2021.785796)

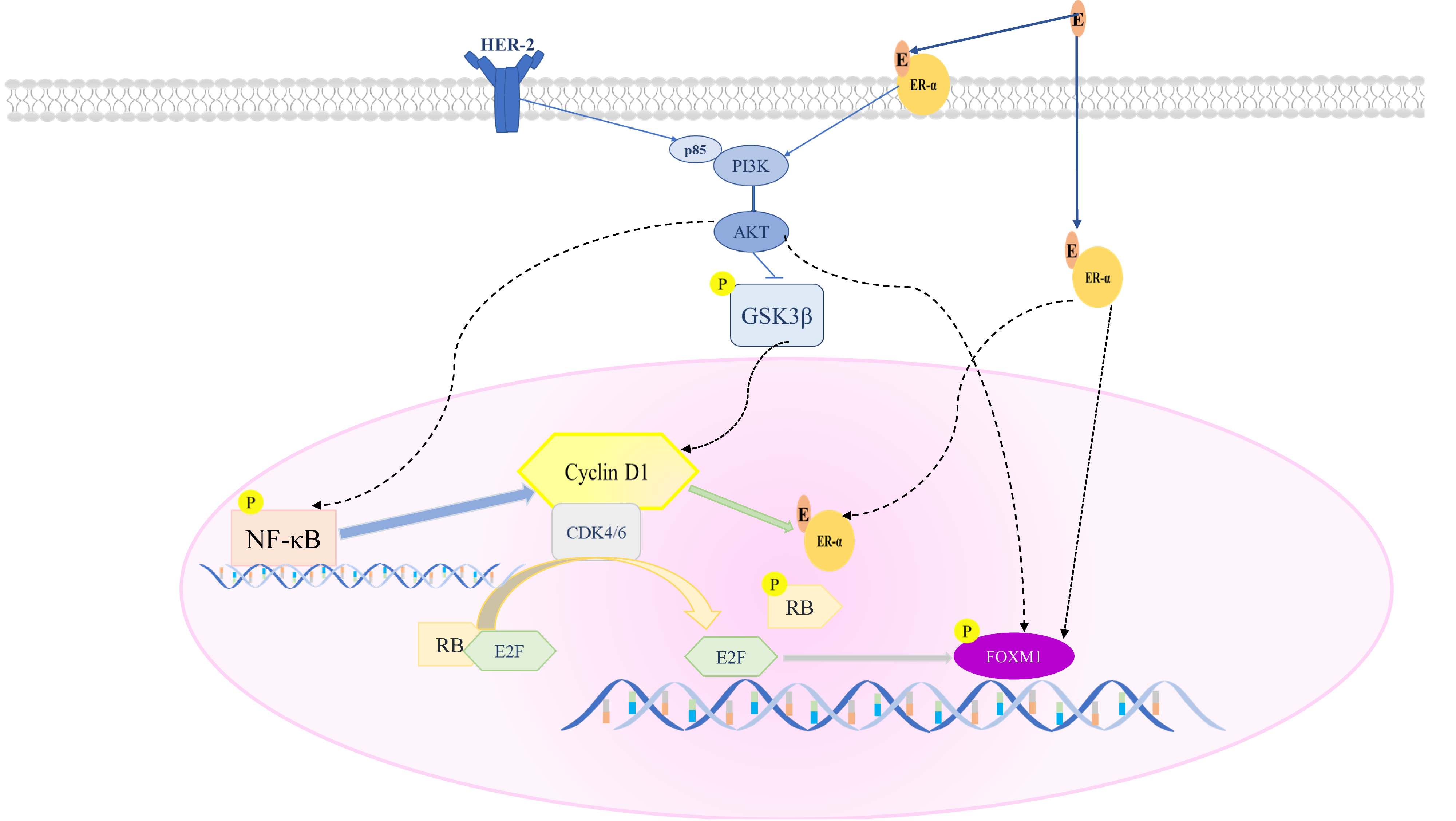

Supplement: Supplementary file 1 [file Image2.TIF]

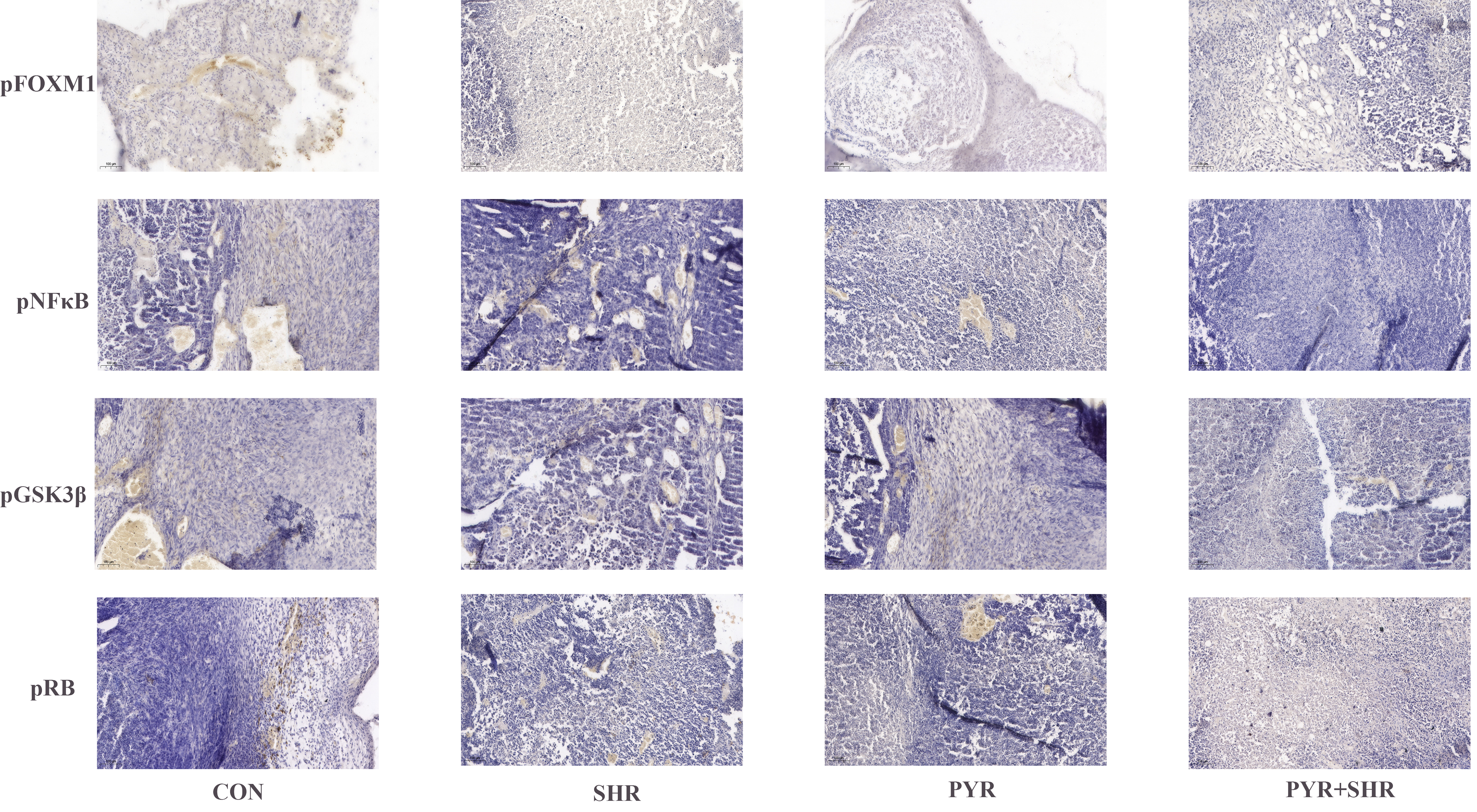

Supplement: Supplementary file 2 [file Image1.TIF]
